# PAMORAs in Cancer and Non-Cancer Opioid-Induced Constipation: A Systematic Review, Meta-Analysis, and Personalized Value-Based Evaluation Integrating Clinical and Patient-Reported Outcomes

**DOI:** 10.3390/jpm16100494

**Published:** 2026-09-24

**Authors:** Francesco Fiorica, Maria Viviana Candela, Jacopo Giuliani, Umberto Tebano, Marta Mandarà, Milena Gabbani, Giuseppe Napoli, Navdeep Singh, Eliana Ivaldi, Paolo Rappa Verona, Nadia Torchio, Chiara Boero, Manuela Cazzulo, Delia Lavagnolo, Francesco Pinta, Marcello Tucci

**Affiliations:** 1 Department of Oncology, Azienda ULSS 9 Scaligera, Via Gianella 1, 37045 Legnago, Italy; 2 Business and Law, High School of Sciences, Luigi Einaudi, 44121 Ferrara, Italy; 3 SS Hospice and Palliative Care, 14100 Asti, Italy; 4 Home-Based Palliative Care, 14100 Asti, Italy; 5 Hospice, Valle Belbo, 14049 Piemonte, Italy; 6 Medical Oncology, Cardinal Massaia Hospital, 14100 Asti, Italy

**Keywords:** opioid-induced constipation, peripherally acting μ-opioid receptor antagonists (PAMORAs), spontaneous bowel movements (SBMs), Patient Assessment of Constipation Quality of Life (PAC-QOL), EQ-5D, health-economic evaluation, value-based pricing

## Abstract

**Background:** Opioid-induced constipation (OIC) is a frequent and clinically relevant complication of long-term opioid therapy in both cancer and non-cancer populations, negatively affecting symptoms, treatment adherence, and health-related quality of life. Peripherally acting μ-opioid receptor antagonists (PAMORAs) represent a mechanism-based therapeutic approach targeting peripheral opioid effects while preserving analgesic activity. However, the overall value of PAMORAs, integrating clinical outcomes, patient-reported benefits, and health-economic implications, has not been comprehensively assessed. **Methods:** A systematic review and meta-analysis of randomized controlled trials (RCTs) comparing PAMORAs versus placebo in adults with OIC was performed. PubMed, Embase, Cochrane CENTRAL, and Web of Science were searched up to June 2025. Studies were stratified according to clinical setting (cancer versus non-cancer populations). The primary outcome was the change in mean weekly spontaneous bowel movements (SBMs). Secondary outcomes included constipation-related quality of life assessed by the Patient Assessment of Constipation–Quality of Life (PAC-QOL) questionnaire and health utility estimation through EQ-5D mapping. A two-step exploratory framework linking SBM improvement to PAC-QOL and subsequently to EQ-5D utilities was applied to extend the economic evaluation across the available evidence base. **Results:** Nineteen RCTs including 5565 patients (2785 PAMORA-treated and 2780 placebo-treated patients) were included. PAMORAs significantly increased weekly SBMs compared with placebo (mean difference [MD] 1.49; 95% CI 1.04–1.95; *p* < 0.0001). The treatment effect was highly consistent in non-cancer populations (MD 1.28; 95% CI 1.10–1.46; I^2^ = 0%) and larger but more heterogeneous in cancer populations (MD 2.67; 95% CI −0.001 to 5.35; I^2^ = 93.7%). In five trials reporting quality-of-life outcomes, PAMORAs were associated with a statistically significant improvement in PAC-QOL scores compared with placebo (MD −0.25; 95% CI −0.41 to −0.10); however, only one study demonstrated an improvement approaching a clinically meaningful threshold, limiting the certainty regarding the clinical relevance of the pooled effect. Mapping analyses provided an exploratory estimate of the relationship between bowel function, patient-reported quality of life, and EQ-5D utilities, allowing estimation of illustrative value-based treatment thresholds. Estimated acceptable monthly treatment costs ranged from approximately €25 to €101 depending on population and trial characteristics. **Conclusions:** PAMORAs consistently improve bowel function in patients with opioid-induced constipation across cancer and non-cancer settings. Available evidence also suggests an improvement in constipation-related quality of life, although the clinical relevance of this effect remains uncertain because of the limited number of studies and the lack of consistently clinically meaningful changes. The exploratory integration of clinical outcomes, patient-reported measures, and health-economic parameters provides a potential framework for personalized treatment evaluation, but the estimated economic thresholds should be interpreted cautiously and should not be considered direct estimates of cross-country cost-effectiveness.

## 1. Introduction

Opioid consumption for the management of cancer-related and non-cancer chronic pain has increased worldwide over the past decades, resulting in a growing population of patients exposed to long-term opioid therapy. In the United States, chronic opioid use has been reported in approximately 5–6% of the general population [1], while opioid consumption in European countries increased by nearly 30% between the 1990s and 2010s [2]. As the number of patients requiring prolonged opioid treatment continues to rise, the prevention and management of opioid-related adverse events represent essential components of patient-centered and individualized supportive care.

Among opioid-related adverse effects, opioid-induced constipation (OIC) is the most frequent gastrointestinal complication associated with long-term opioid therapy [3]. OIC affects approximately 40–80% of opioid-treated patients and results from activation of peripheral μ-opioid receptors within the gastrointestinal tract, leading to impaired intestinal motility, increased fluid absorption, and enhanced sphincter tone [3,4]. Beyond its impact on bowel function, OIC significantly affects physical and psychological well-being, treatment adherence, and overall health-related quality of life (HRQoL), representing a substantial burden for both patients and healthcare systems [5].

Current guidelines recommend a multimodal approach for OIC management, combining lifestyle interventions, conventional laxatives, and targeted pharmacological treatments [6,7]. However, lifestyle modifications, including exercise, dietary interventions, and adequate hydration, may be difficult to implement in specific patient populations, such as elderly individuals, frail patients, or those with advanced cancer. Conventional laxatives remain widely used, although their efficacy may be limited by their nonspecific mechanism of action. In contrast, peripherally acting μ-opioid receptor antagonists (PAMORAs), including naloxegol, methylnaltrexone, and naldemedine, represent a mechanism-based therapeutic approach by selectively antagonizing peripheral gastrointestinal μ-opioid receptors while preserving central analgesic activity [6,7,8].

Despite their established efficacy and favorable safety profile, PAMORAs remain underused in routine clinical practice, and OIC continues to be frequently underdiagnosed and undertreated [9]. This therapeutic gap highlights the need not only to improve awareness and implementation of evidence-based treatments but also to identify which patients are most likely to derive meaningful clinical benefit from targeted interventions. In this context, the management of OIC represents an important area of personalized supportive care, where treatment decisions should integrate disease characteristics, clinical setting, symptom burden, patient priorities, and expected impact on quality of life.

Traditional efficacy outcomes, such as changes in spontaneous bowel movements (SBMs), provide essential information regarding treatment activity but may not fully capture the individual burden of OIC or the overall benefit perceived by patients. Patient-reported outcome measures (PROMs), including the Patient Assessment of Constipation–Quality of Life (PAC-QOL) questionnaire [10], allow a more comprehensive assessment of treatment impact by evaluating physical, psychosocial, and functional consequences of constipation. Similarly, generic health utility instruments such as the EQ-5D [11] enable the translation of quality-of-life improvements into quality-adjusted life years (QALYs), supporting value-based healthcare decisions.

To date, the relationship between objective clinical outcomes, patient-reported benefits, and health-economic value has not been systematically explored across the PAMORA trial landscape. A comprehensive integration of these domains may provide a more personalized framework for evaluating treatment effectiveness and supporting clinical decision-making across different patient populations, particularly considering the relevant differences between cancer and non-cancer settings.

The present systematic review and meta-analysis was therefore designed with two complementary objectives: (1) to provide an updated synthesis of the efficacy of PAMORAs in patients with cancer and non-cancer OIC, integrating bowel function outcomes and patient-reported quality-of-life measures; and (2) to extend the evidence base into the health-economic domain by mapping clinical and quality-of-life outcomes to EQ-5D utilities and estimating exploratory value-based treatment thresholds according to established willingness-to-pay criteria. By combining clinical efficacy, patient-centered outcomes, and economic evaluation, this study aims to provide a comprehensive framework to support personalized therapeutic strategies for the management of opioid-induced constipation.

## 2. Materials and Methods

### 2.1. Study Design and Search Strategy

This systematic review and meta-analysis was conducted according to a predefined analytical framework including: (1) literature search and study selection; (2) eligibility assessment and data extraction; (3) synthesis of clinical outcomes; (4) evaluation of patient-reported outcomes and utility mapping; and (5) health-economic analysis.

The study was designed to evaluate the clinical and exploratory economic value of peripherally acting μ-opioid receptor antagonists (PAMORAs) in opioid-induced constipation (OIC), considering the potential differences between cancer and non-cancer populations. The analytical approach aimed to integrate objective clinical outcomes, patient-reported outcomes, and health utility measures to support a more individualized evaluation of treatment benefit.

A systematic review and meta-analysis of randomized controlled trials (RCTs) evaluating PAMORAs for OIC was performed. The review protocol was registered in the PROSPERO database (ID 1139923) and the study was conducted according to the Preferred Reporting Items for Systematic Reviews and Meta-Analyses (PRISMA) 2020 guidelines [12].

Eligible studies were identified through systematic searches of published literature and conference proceedings, without language restrictions. We included RCTs comparing PAMORAs with placebo in adult patients receiving opioid therapy for either cancer-related pain or chronic non-cancer pain.

The complete search strategies for each database, including the search terms, Boolean operators, filters, and the date of the final search, are provided in Supplementary Table S1. The search was conducted in PubMed, Embase, Cochrane CENTRAL, and Web of Science up to June 2025.

### 2.2. Study Selection Criteria and Data Extraction

Studies were considered eligible if they evaluated adult patients with OIC and reported outcomes that allowed quantitative assessment of bowel function, expressed as changes in mean spontaneous bowel movements (SBMs) per week.

Trials were included when SBM outcomes were directly reported or could be reliably reconstructed from available published data. In addition, data regarding patient-reported outcomes were extracted, including constipation-related quality of life measured by the Patient Assessment of Constipation–Quality of Life (PAC-QOL) questionnaire and, when available, generic health-related quality of life utilities assessed using the EQ-5D instrument.

The PAC-QOL is a validated 28-item patient-reported outcome measure assessing physical discomfort, psychosocial impact, worries/concerns, and satisfaction related to constipation. Scores range from 0 to 4, with lower scores indicating better quality of life.

Considering the clinical heterogeneity between cancer and non-cancer populations, studies were categorized according to the predominant clinical setting. Trials were classified as cancer or non-cancer populations when at least 60% of enrolled patients belonged to the respective subgroup. This stratification was performed to explore whether treatment effects and patient-centered outcomes differed according to clinical context.

Data extraction was independently performed by two reviewers, and discrepancies were resolved through consensus. Information regarding study characteristics, patient populations, interventions, comparators, clinical outcomes, quality-of-life measures, and economic parameters was collected.

Baseline demographic characteristics, including age, sex, opioid exposure, and relevant disease characteristics, were extracted and summarized for each included study. Where reported, age was presented as mean ± standard deviation or median with range/interquartile range. Because reporting formats varied across studies, these data were not considered sufficient to establish complete baseline comparability when information was unavailable or inconsistently reported.

The economic evaluation component was reported according to the Consolidated Health Economic Evaluation Reporting Standards (CHEERS) 2022 recommendations [13].

### 2.3. Meta-Analysis of Clinical Outcomes

The primary clinical outcome was the change in mean weekly spontaneous bowel movements (SBMs) between PAMORA-treated patients and placebo-treated controls.

Treatment effects were expressed as mean differences (MDs) with corresponding 95% confidence intervals (CIs) [14,15]. Pooled estimates were calculated using random-effects models based on the inverse variance method with restricted maximum likelihood estimation for τ^2^. Statistical heterogeneity was assessed using the I^2^ statistic.

A random-effects model was prespecified because clinically relevant heterogeneity was expected across the included trials, including differences in patient populations, cancer versus non-cancer clinical settings, opioid exposure, treatment characteristics, follow-up duration, and study design. Accordingly, we did not assume that all studies estimated one common underlying treatment effect. The random-effects model allows the underlying true treatment effect to vary between studies and incorporates both within-study uncertainty and between-study heterogeneity into the pooled estimate. Restricted maximum likelihood (REML) was used to estimate the between-study variance (τ^2^).

Risk of bias was independently assessed by two reviewers (FF, MT) using the Cochrane Risk of Bias tool [16]. The evaluation included seven domains: random sequence generation, allocation concealment, blinding of participants and personnel, blinding of outcome assessment, incomplete outcome data, selective reporting, and other potential sources of bias. The detailed study-level risk of bias assessments across these domains are summarized in Supplementary Table S2.

Study-level assessments were applied for domains related to randomization, allocation concealment, selective reporting, and other biases, whereas outcome-level assessments were applied for blinding and incomplete outcome data. Disagreements were resolved through consultation with a third investigator.

Publication bias and small-study effects were visually assessed using funnel plots for the primary endpoint (weekly SBMs; Supplementary Figure S1). An exploratory funnel plot was also generated for the secondary quality-of-life endpoint (PAC-QOL; Supplementary Figure S2), though its interpretation is limited by the small number of eligible studies (n = 5).

Predefined subgroup analyses were performed separately for cancer and non-cancer populations to explore potential differences in treatment effect according to clinical setting.

Forest plots were generated for the overall analysis and prespecified cancer/non-cancer subgroup analyses to display study-specific effect estimates with 95% confidence intervals, together with the corresponding pooled estimates.

### 2.4. Quality-of-Life Analysis and Utility Mapping

To incorporate patient-centered outcomes into the evaluation framework, quality-of-life improvements were analyzed using validated patient-reported outcome measures.

For studies reporting PAC-QOL and EQ-5D simultaneously [17,18], an internal mapping analysis was performed to quantify the relationship between constipation-specific quality of life and generic health utilities [19]. A study-level regression model was developed to estimate the association between PAC-QOL improvement and EQ-5D utility gain.

The derived mapping function was subsequently applied to studies reporting PAC-QOL without direct EQ-5D measurements, allowing estimation of health utilities across a broader evidence base.

Because the mapping analyses were based on aggregate study-level data rather than individual patient-level observations, the resulting utility estimates were considered exploratory. Potential ecological bias and uncertainty in the cross-walk were therefore explicitly considered when interpreting the economic results.

### 2.5. SBM-to-Utility Mapping

Because spontaneous bowel movements represented the most frequently reported endpoint across PAMORA trials, a secondary mapping analysis was performed to extend the health-economic evaluation to studies lacking direct quality-of-life measurements.

A regression model was developed using trials reporting both SBM changes and PAC-QOL improvements, generating a quantitative relationship between bowel function improvement and constipation-related quality of life.

This relationship was subsequently integrated with the PAC-QOL-to-EQ-5D mapping function, generating a two-step framework:

### 2.6. SBM Improvement → PAC-QOL Improvement → EQ-5D Utility Gain

This approach enabled translation of objective clinical outcomes into patient-centered and economic outcomes across the entire evidence base, including trials reporting only SBM endpoints.

Given the study-level nature of both mapping steps, this framework was considered exploratory and was intended to provide an indirect estimate rather than a substitute for direct patient-level utility measurement.

### 2.7. Health-Economic Evaluation

Estimated EQ-5D utilities were converted into quality-adjusted life years (QALYs) over the duration of each trial observation period.

QALYs were subsequently monetized using a willingness-to-pay threshold of €30,000 per QALY, allowing estimation of illustrative value-based monthly treatment thresholds for PAMORA therapy.

The economic analysis was designed to integrate clinical benefit, quality-of-life improvement, and resource value, providing an additional framework for evaluating personalized treatment strategies in patients with opioid-induced constipation.

Because the included studies were conducted in different countries and healthcare systems, reported costs may differ according to currency, price levels, inflation, reimbursement structures, healthcare expenditure, and purchasing power. Where the primary studies did not provide sufficient information regarding the cost year, costing perspective, currency, and resource components, a fully harmonized cross-country cost analysis was not considered sufficiently reliable. Therefore, the estimated monthly thresholds were interpreted as exploratory estimates rather than directly comparable country-independent measures of cost-effectiveness. Ideally, cross-country cost comparisons should use a common price year, a common currency, appropriate inflation adjustment, and purchasing-power-parity (PPP) adjustment where appropriate.

## 3. Results

### 3.1. Study Selection and Characteristics

A total of 19 randomized controlled trials (RCTs) were included in the meta-analysis, comprising 5565 patients overall (2785 treated with PAMORAs and 2780 receiving placebo). The included studies evaluated different PAMORA agents, including naloxegol, naldemedine, and methylnaltrexone, in patients with opioid-induced constipation (OIC) receiving opioid therapy for either non-cancer pain or cancer-related conditions.

The study selection process is summarized in the PRISMA flow diagram (Figure 1). A total of 1240 records were identified through database and additional searches. After removal of 380 duplicates, 860 records were screened by title and abstract, of which 795 were excluded. Full texts were assessed for 65 records, with 46 excluded for the following reasons: non-RCT or observational study design (n = 18); SBM outcomes missing or not reconstructible (n = 14); non-eligible intervention or lack of a placebo comparator (n = 9); and duplicate reporting or interim analysis (n = 5). Nineteen studies were ultimately included in the quantitative synthesis.

Most studies were conducted in patients with chronic non-cancer pain receiving long-term opioid therapy (14 trials) [18,20,21,22,23,24,25,26,27,28,29,30,31], whereas a smaller number focused on patients with advanced cancer (5 trials) [17,18,32,33,34]. Sample sizes varied considerably, ranging from fewer than 100 participants in exploratory studies [28,33] to more than 1200 patients in large phase III clinical programs.

The primary endpoint across studies was the change in weekly spontaneous bowel movements (SBMs), evaluated over treatment periods ranging from 14 days in cancer populations to 12 weeks in non-cancer populations. Secondary endpoints included responder rates (defined as ≥3 SBM/week and an increase from baseline), time to first SBM, and rescue laxative use.

Importantly, a subset of trials collected patient-reported outcomes, including the Patient Assessment of Constipation–Quality of Life (PAC-QOL) questionnaire and, in selected studies, EQ-5D utility measurements. These data allowed integration of symptomatic improvement with patient-perceived benefit and health utility evaluation.

Baseline characteristics of the included populations are summarized in Table 1, including age, sex distribution, opioid exposure, and relevant clinical characteristics. Age varied across studies, with reported values ranging from 20 to 87 years/mean or median values of 48.3–67.8, where available. Because several studies reported demographic variables using different formats and some characteristics were incompletely reported, the populations should be considered broadly comparable with respect to the available baseline characteristics rather than strictly equivalent.

### 3.2. Change in Spontaneous Bowel Movements (SBMs)

The pooled analysis demonstrated that PAMORAs significantly increased the number of spontaneous bowel movements compared with placebo, with a mean difference (MD) of 1.49 SBMs per week (95% CI 1.04–1.95; *p* < 0.0001) (Figure 1).

Overall, the included trials demonstrated high methodological quality, with 16 out of 19 studies presenting a low overall risk of bias (Supplementary Table S2). Visual inspection of the funnel plot revealed asymmetry (Supplementary Figure S1), which was formally confirmed by Egger’s regression test (z = 5.0343, *p* < 0.0001). The limit estimate as standard error approached zero was b = 0.3747 (95%CI: −0.0416–0.7910).

The corresponding forest plot (Figure 2) displays the individual study estimates and 95% confidence intervals, as well as the pooled random-effects estimate.

Between-study heterogeneity was substantial (I^2^ = 83.3%), reflecting variability among trials and differences in clinical populations, treatment settings, and study designs.

In the non-cancer population (14 studies), PAMORA therapy showed a consistent and reproducible benefit, with an MD of 1.28 SBMs per week (95% CI 1.10–1.46) and no relevant heterogeneity (I^2^ = 0%). These findings indicate a highly consistent treatment effect across studies involving patients with chronic non-cancer pain.

In cancer populations (5 studies), the estimated treatment effect was numerically greater (MD 2.67; 95% CI −0.001 to 5.35), although associated with substantial heterogeneity (I^2^ = 93.7%), resulting in reduced precision of the pooled estimate. The wide confidence interval and high heterogeneity indicate substantial uncertainty around the magnitude of the pooled effect in this subgroup.

The test for subgroup differences did not demonstrate a statistically significant interaction between cancer and non-cancer populations (Q = 2.07, df = 1; *p* = 0.15).

At the individual drug level, both naldemedine and naloxegol demonstrated superiority compared with placebo in improving weekly SBM frequency.

Naldemedine showed consistent efficacy in non-cancer populations, with mean differences ranging between +1.0 and +1.4 SBMs per week compared with placebo (COMPOSE-1: MD 1.30; 95% CI 0.77–1.83; COMPOSE-2: MD 1.40; 95% CI 0.89–1.91). In cancer populations, naldemedine also improved bowel function, as observed in the Hamano trial (MD 0.41; 95% CI 0.23–0.58) and the Katakami study (MD 3.25; 95% CI 1.82–4.68), although with considerable variability among studies.

Naloxegol demonstrated improvements of approximately one additional SBM per week compared with placebo (KODIAC-04: MD 1.00; 95% CI 0.50–1.50; KODIAC-05: MD 1.04; 95% CI 0.54–1.54), with relatively homogeneous results across trials.

Overall, PAMORAs demonstrated a consistent improvement in bowel function, particularly in non-cancer populations. The larger but substantially more heterogeneous estimate observed in cancer populations should be interpreted cautiously, and the available evidence does not establish a statistically significant difference in treatment effect between cancer and non-cancer settings.

### 3.3. Change in Quality of Life

Five randomized trials reported quality-of-life outcomes using the Patient Assessment of Constipation–Quality of Life (PAC-QOL) questionnaire.

In the pooled analysis, PAMORA treatment was associated with a statistically significant improvement compared with placebo (MD −0.26; 95% CI −0.42 to −0.10; *p* = 0.01) (Figure 3).

Although statistically significant, the pooled effect should be interpreted in the context of the limited number of contributing studies and the uncertainty regarding its clinical relevance.

Heterogeneity among studies was moderate (I^2^ = 56%; 95% CI 0–83.7%; *p* = 0.06). The greatest improvement was observed in the Hamano trial conducted in patients with cancer (MD −0.49; 95% CI −0.73 to −0.25). This was the only study in the available evidence base showing an improvement that approached or exceeded a commonly cited threshold for clinically meaningful change in PAC-QOL. Therefore, the pooled statistically significant effect should not be interpreted as demonstrating a consistently clinically meaningful improvement in quality of life across populations.

Non-cancer trials, including COMPOSE-3 (MD −0.30) and KODIAC-04 (MD −0.12), also demonstrated improvements, although with smaller effect sizes.

Overall, the available evidence suggests that PAMORA therapy may improve constipation-related quality of life; however, the magnitude and clinical relevance of this benefit remain uncertain because only five trials reported PAC-QOL and clinically meaningful improvement was not consistently demonstrated across studies.

Visual inspection of the exploratory funnel plot for PAC-QOL scores showed no obvious asymmetry (Supplementary Figure S2). Egger’s regression test confirmed the absence of significant funnel plot asymmetry (z = −0.8218, *p* = 0.4112; limit estimate b = −0.1675, 95% CI: −0.3142 to −0.0209), indicating no evident publication bias for this endpoint.

### 3.4. Health-Economic Evaluation

To estimate the economic value of PAMORA therapy, improvements in PAC-QOL were translated into EQ-5D utilities using a study-level mapping approach based on trials reporting both outcomes [17,18].

The analysis showed that each one-point improvement in PAC-QOL corresponded to an average gain of approximately 0.09 EQ-5D utility units (Table 2). Estimated utilities were subsequently converted into QALYs over the trial observation periods (12 weeks in non-cancer populations and 14 days in cancer populations) and monetized using a willingness-to-pay threshold of €30,000 per QALY.

Under these assumptions, the estimated acceptable monthly treatment costs for PAMORAs were approximately €52 in pooled non-cancer populations, €62 in COMPOSE-3, €25 in KODIAC-04 [18], €37 in KODIAC-05 [18], and €101 per 28 days in the cancer setting [17].

All estimates were standardized to monthly costs (28–30 days of therapy) to allow comparison across trials with different follow-up durations.

These estimates should be interpreted as exploratory value-based thresholds rather than direct estimates of cost-effectiveness. In particular, monthly treatment costs reported across countries may not be directly comparable because of differences in currency, inflation, healthcare price levels, reimbursement policies, healthcare expenditure, and purchasing power. A fully standardized cross-country analysis would ideally require adjustment to a common price year and currency, together with appropriate PPP adjustment and harmonization of the costing perspective and included resource components. Such harmonization was not consistently possible from the published trial data.

The estimates therefore primarily illustrate how the modeled clinical and utility gains translate into hypothetical willingness-to-pay thresholds under the specified assumptions, rather than representing universally applicable acceptable prices.

To extend the economic analysis across the complete evidence base, a secondary mapping procedure was performed using SBM outcomes reported in all pivotal PAMORA trials.

In studies reporting both SBM changes and PAC-QOL improvement, study-level regression demonstrated a strong association between bowel function improvement and quality-of-life gain. The fitted model showed a slope of approximately +0.48 PAC-QOL points per additional SBM per week, an intercept of −0.68, and explained variance of R^2^ = 0.80 (Figure 4).

Because this regression was based on study-level rather than individual patient-level data, the strength of the observed association should be interpreted cautiously and does not establish a causal relationship between SBM improvement and quality-of-life improvement.

PAC-QOL improvements were subsequently mapped onto EQ-5D utilities using the study-level PAC-QOL-to-EQ-5D relationship derived from trials reporting both outcomes, corresponding to an estimated gain of 0.08–0.10 EQ-5D utility points per PAC-QOL point.

This two-step framework (SBM → PAC-QOL → EQ-5D) enabled an exploratory harmonization of clinical, patient-reported, and economic outcomes across the complete PAMORA evidence base. By linking objective symptom improvement with patient-perceived benefit and health utility, the model provided a potential framework for evaluating the value of PAMORA therapy across heterogeneous patient populations (Figure 5).

## 4. Discussion

This systematic review and meta-analysis demonstrates that peripherally acting μ-opioid receptor antagonists (PAMORAs) provide a consistent improvement in bowel function in patients with opioid-induced constipation (OIC), with evidence of improvement in patient-reported quality of life that is statistically significant but of uncertain clinical relevance. Importantly, the present study extends previous efficacy-focused analyses by integrating clinical outcomes, patient-centered measures, and an exploratory health-economic framework into a unified framework.

PAMORAs were effective in both cancer and non-cancer populations, although differences in the magnitude and consistency of treatment effects emerged according to clinical setting. The non-cancer population showed a highly homogeneous improvement in bowel function, whereas cancer studies demonstrated larger but more heterogeneous effects. This variability likely reflects the complexity of patients with advanced cancer, where constipation burden may be influenced by multiple factors, including disease progression, concomitant medications, nutritional status, opioid exposure, and overall symptom burden. These considerations may contribute to the observed heterogeneity and support cautious interpretation of the larger pooled estimate in cancer populations.

A major contribution of this study is the integration of patient-reported outcomes into the assessment of PAMORA effectiveness. While improvement in bowel frequency remains an essential clinical endpoint, the burden of OIC extends beyond stool frequency and includes physical discomfort, psychological distress, functional impairment, and reduced quality of life. The statistically significant pooled improvement in PAC-QOL suggests that PAMORA therapy may translate into patient-perceived benefits; however, only one of the five studies reporting PAC-QOL demonstrated an improvement approaching or exceeding a clinically meaningful threshold. Therefore, the available evidence is insufficient to conclude that PAMORAs consistently produce a clinically meaningful improvement in constipation-related quality of life.

Previous meta-analyses of PAMORAs [29,30,36,37,38,39] have primarily focused on efficacy and safety outcomes, demonstrating consistent improvements in bowel function compared with placebo. More recent evidence has increasingly emphasized patient-reported outcomes and constipation-related quality of life, including analyses of the COMPOSE program showing significant improvements in PAC-QOL and PAC-SYM with naldemedine [40]. Recent systematic reviews have also expanded the evidence base in patients with cancer, although heterogeneity across interventions, populations, and outcome definitions remains relevant [30,41]. However, these analyses have rarely addressed the relationship between clinical improvement, patient-reported benefit, and economic value. First, we applied a consistent analytical framework across cancer and non-cancer populations, allowing exploration of potential differences according to clinical context. Second, we incorporated patient-reported outcomes through PAC-QOL assessment, providing additional information regarding the patient-reported dimension of treatment benefit. Third, we developed an exploratory mapping framework linking SBM improvement to PAC-QOL and subsequently to EQ-5D utilities, enabling translation of symptomatic improvement into health-economic outcomes.

The two-step mapping approach (SBM → PAC-QOL → EQ-5D) represents a pragmatic strategy to overcome a common limitation of clinical trial evidence: the absence of direct utility measurements in many studies. Because SBM was consistently reported as a primary endpoint across PAMORA trials, this approach allowed integration of a broader evidence base into an economic framework. The observed association between improvement in bowel frequency and better quality of life supports the potential clinical relationship between these domains, although the relationship should be interpreted cautiously given the study-level nature of the analysis, the limited number of studies contributing to the mapping models, and the possibility of ecological bias.

The economic evaluation suggested that PAMORA therapy may provide value under the assumptions used in the present model. Importantly, these estimates should be interpreted as exploratory because they depend on the assumed SBM-to-PAC-QOL and PAC-QOL-to-EQ-5D relationships, the selected willingness-to-pay threshold, and the short observation periods of the underlying trials.

An additional limitation concerns the comparability of costs across countries. Monthly expenditure may reflect not only the price of PAMORA treatment but also country-specific healthcare price levels, inflation, currency valuation, reimbursement systems, patient co-payments, and purchasing power. Consequently, standardizing reported costs only by converting them to a common currency or by expressing them on a monthly basis does not eliminate cross-country measurement differences. Ideally, future economic analyses should harmonize costs to a common price year and currency, apply appropriate inflation and PPP adjustments, and use a consistent costing perspective. Where these data are unavailable, pooled or cross-country cost thresholds should be interpreted primarily as illustrative rather than directly transferable estimates.

The present analysis also does not account for potential differences in affordability between patients or healthcare systems. A treatment cost that may be considered acceptable under a fixed willingness-to-pay framework may represent substantially different financial burdens across settings. Future studies should therefore distinguish between healthcare-system cost-effectiveness and patient-level affordability when evaluating the value of PAMORA therapy.

From a personalized medicine perspective, these findings support a more individualized approach to OIC management. Recent expert recommendations have emphasized the importance of systematic assessment of OIC severity, regular monitoring of treatment response, and timely escalation to mechanism-based therapies such as PAMORAs when conventional laxatives are insufficient [2,42]. Patients receiving opioids represent a clinically heterogeneous population, including individuals with chronic non-cancer pain, advanced cancer, different opioid regimens, and variable baseline symptom burden. The integration of objective bowel function measures, patient-reported outcomes, and economic considerations could contribute to identifying patients most likely to derive meaningful benefit from PAMORA therapy and optimize allocation of healthcare resources.

This meta-analysis has several strengths. First, it provides an integrated evaluation of PAMORA therapy across different clinical settings, combining traditional efficacy outcomes with patient-centered and economic endpoints. Second, the mapping strategy allowed inclusion of trials that would otherwise have been excluded from economic analyses due to the absence of direct utility measurements. Third, the comparison between cancer and non-cancer populations provides clinically relevant information for treatment interpretation in different patient groups. Fourth, the use of random-effects meta-analysis and presentation of study-specific estimates through forest plots provides an explicit assessment of between-study uncertainty and heterogeneity.

Several limitations should also be acknowledged. First, direct PAC-QOL and EQ-5D data were available only from a limited number of studies, requiring the use of mapping approaches to estimate utilities in the remaining trials. Because these analyses were based on aggregate study-level data, ecological bias cannot be excluded. Individual patient-level analyses would provide a more accurate assessment of the relationship between symptom improvement, quality of life, and utility gain.

Second, most included trials had relatively short follow-up periods, generally ranging from two to twelve weeks, limiting evaluation of long-term effectiveness and sustainability of benefit. Third, substantial heterogeneity was observed, particularly among cancer studies, reducing the precision of pooled estimates in this subgroup.

Fourth, linear regression testing revealed statistically significant funnel plot asymmetry for the primary endpoint of SBM change (z = 5.0343, *p* < 0.0001), suggesting potential publication bias or small-study effects. This asymmetry was largely driven by small oncology trials exhibiting disproportionately large effect estimates, whereas secondary outcomes such as PAC-QOL demonstrated no evident asymmetry (z = −0.8218, *p* = 0.4112).

Fifth, although the pooled PAC-QOL effect was statistically significant, only one study showed an improvement approaching or exceeding a clinically meaningful threshold, and therefore the clinical relevance of the pooled effect remains uncertain.

Sixth, the economic analysis is subject to uncertainty arising from cross-country differences in costs and affordability. The available publications did not consistently provide the information required for complete harmonization of costs by price year, currency, healthcare purchasing power, and costing perspective. Therefore, the estimated monthly thresholds should not be interpreted as directly comparable international cost-effectiveness estimates.

Finally, the economic model assumed a relatively stable relationship between clinical improvement and utility gain, which may vary according to disease severity, patient characteristics, and healthcare setting.

These findings are broadly consistent with the growing contemporary literature supporting the efficacy of PAMORAs while also highlighting the importance of patient-centered outcomes in evaluating treatment benefit [9,31,34,35]. Recent evidence suggests that improvements in bowel function may be accompanied by improvements in constipation-related symptoms and quality of life, although the availability of direct patient-reported and utility data remains limited. This gap supports the need for integrated approaches such as the exploratory framework proposed in the present study.

Despite these limitations, this study provides a potential exploratory framework integrating clinical efficacy, patient experience, and economic considerations in the evaluation of PAMORA therapy. By moving beyond symptom-based outcomes and incorporating patient-centered measures, the findings may contribute to a more personalized approach to supportive care in patients receiving opioid therapy.

## 5. Conclusions

In this systematic review and meta-analysis, PAMORAs demonstrated consistent efficacy in improving bowel function in both cancer and non-cancer populations receiving opioid therapy. PAMORA treatment was also associated with a statistically significant improvement in constipation-related quality of life; however, the clinical relevance of this improvement remains uncertain because clinically meaningful changes were not consistently demonstrated across the available studies.

By integrating clinical outcomes, patient-reported measures, and an exploratory health-economic framework, our study provides a comprehensive approach for assessing the potential value of PAMORA treatment. The estimated value-based monthly thresholds should be interpreted cautiously, particularly because of uncertainty in the mapping analyses and differences in healthcare costs, purchasing power, and affordability across countries.

The findings support the role of PAMORAs as targeted therapeutic options within personalized supportive care pathways, particularly in patients experiencing clinically relevant opioid-induced constipation despite conventional management. Future studies incorporating long-term follow-up, standardized cross-country economic methods, real-world resource utilization, and individual patient-level utility analyses will further refine the identification of patients most likely to benefit from these treatments.

## Figures and Tables

**Figure 1:**
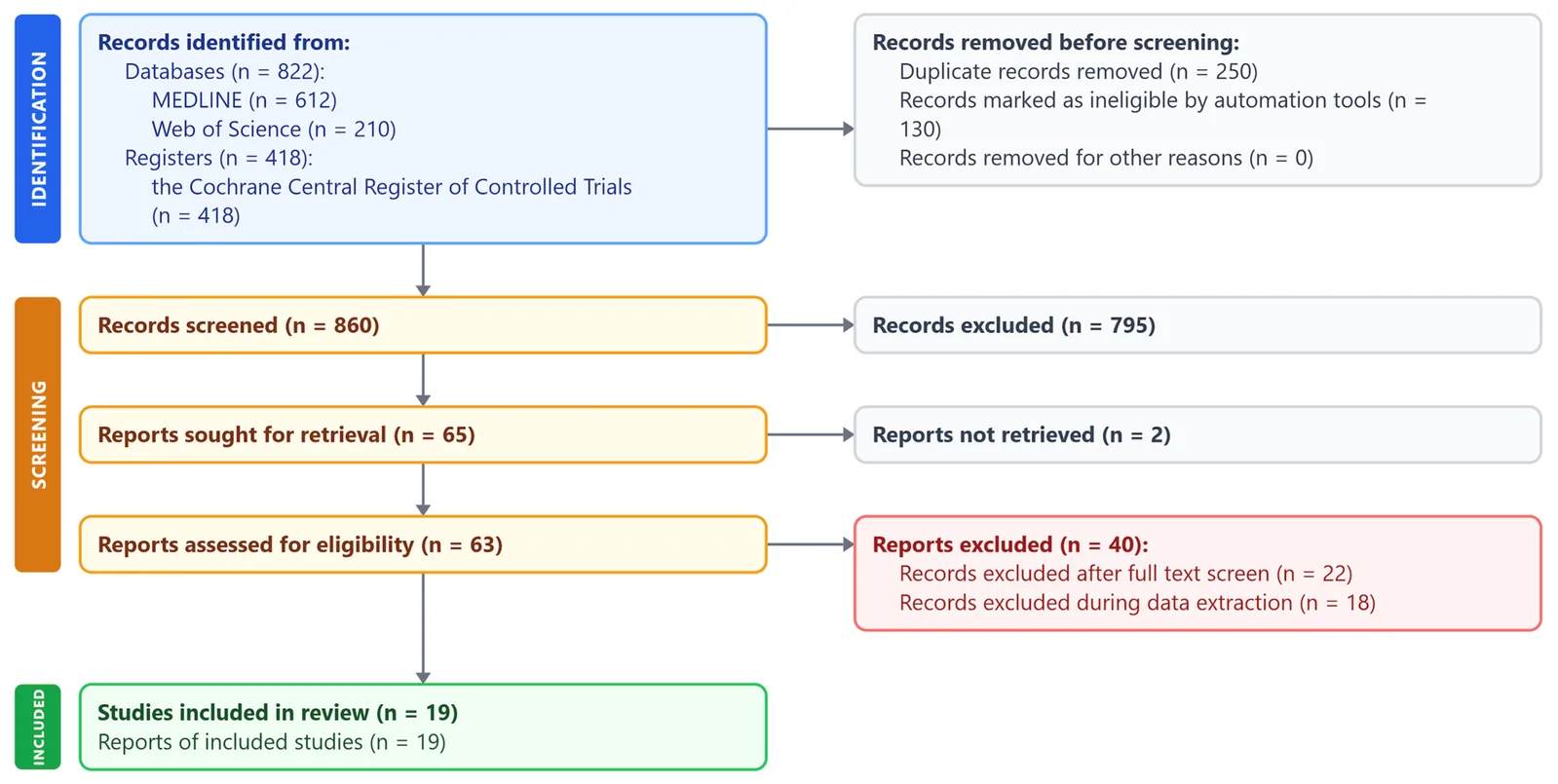
PRISMA flow diagram illustrating the study selection process.

**Figure 2:**
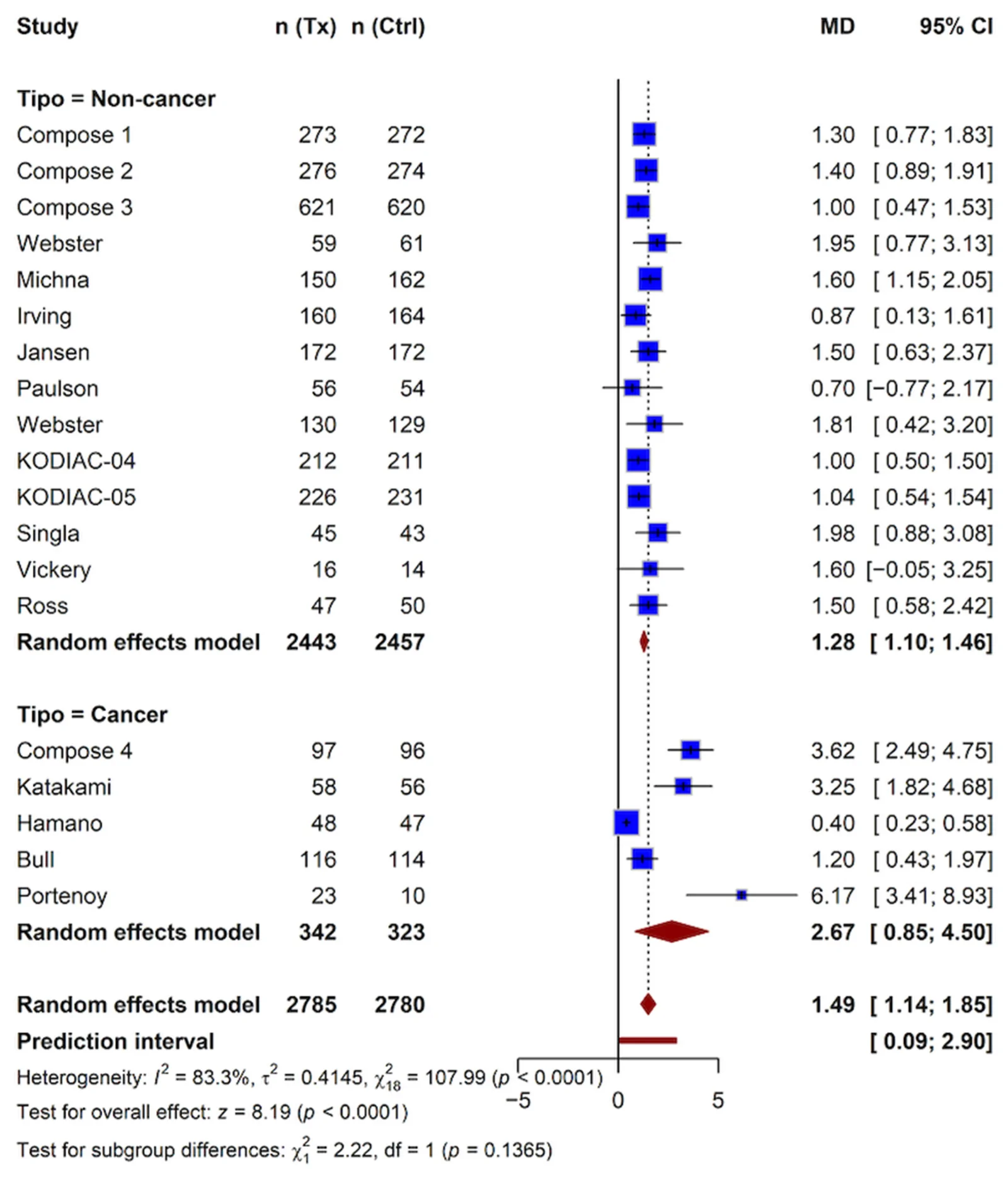
Forest plot of the effect of PAMORAs versus placebo on weekly spontaneous bowel movements (SBMs). Results are stratified by population type (non-cancer vs. cancer). Diamonds represent pooled random-effects estimates with 95% confidence intervals [17,18,20,21,22,23,24,25,26,27,28,29,31,32,33,34,35].

**Figure 3:**
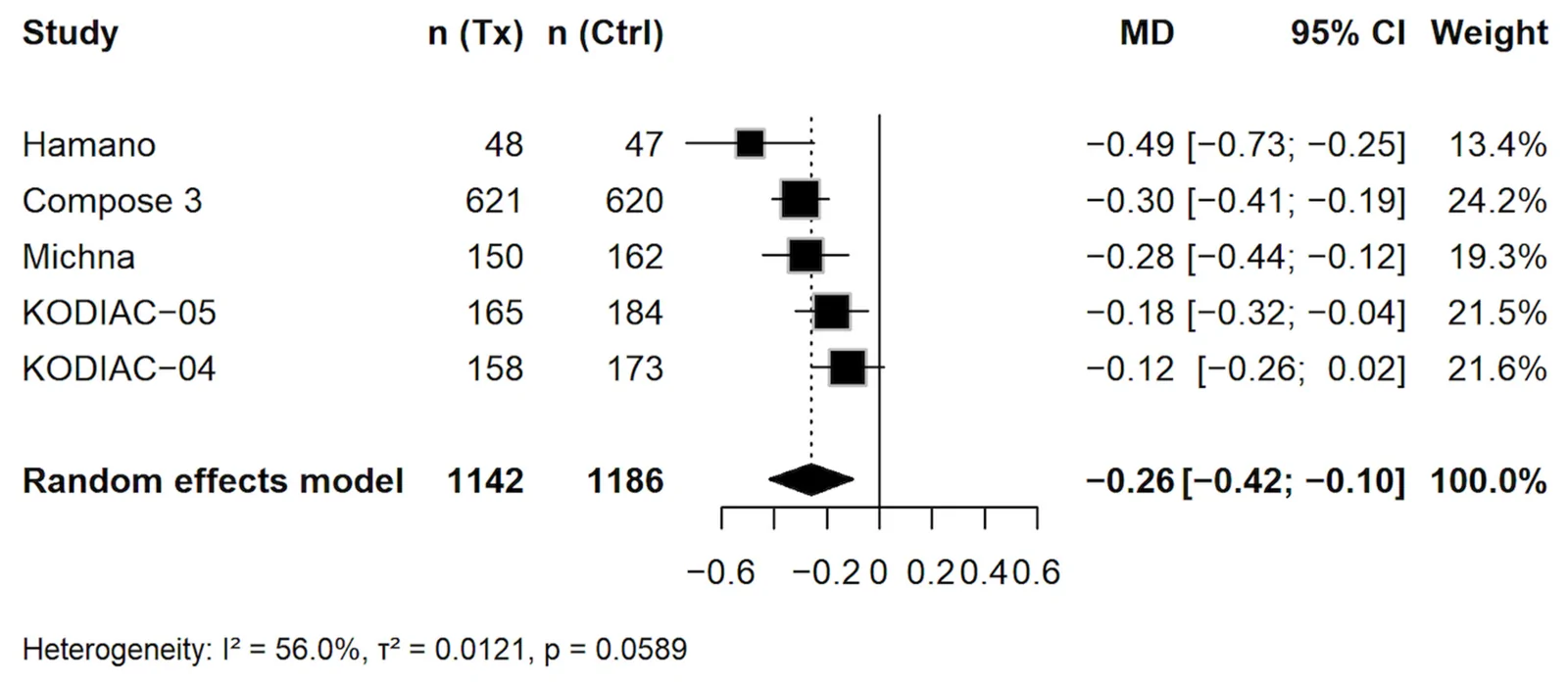
Forest plot of mean difference (MD) in PAC-QOL scores between PAMORAs and placebo. Negative values indicate improvement in quality of life. Diamonds represent pooled random-effects estimates with 95% confidence intervals [17,18,26,27].

**Figure 4:**
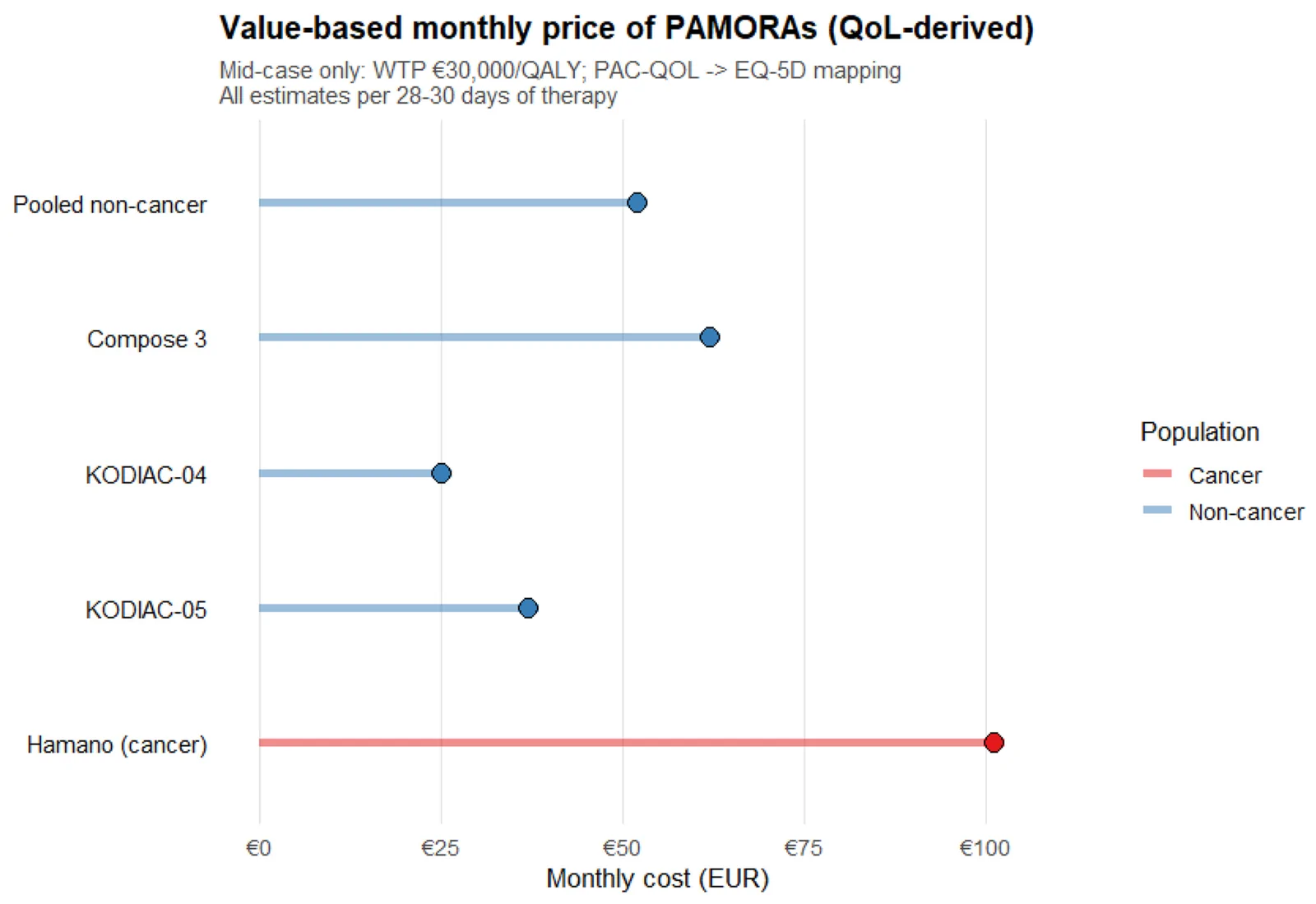
Value-based monthly cost estimates for PAMORAs derived from quality-of-life improvements measured with PAC-QOL and mapped to EQ-5D utilities. Results are shown for pooled non-cancer and cancer populations, as well as for individual pivotal trials. Estimates are based on a willingness-to-pay threshold of €30,000 per QALY and expressed per 28–30 days of therapy. Dots indicate mid-case estimates, while bars represent the corresponding monthly value-based price [17,18,27].

**Figure 5:**
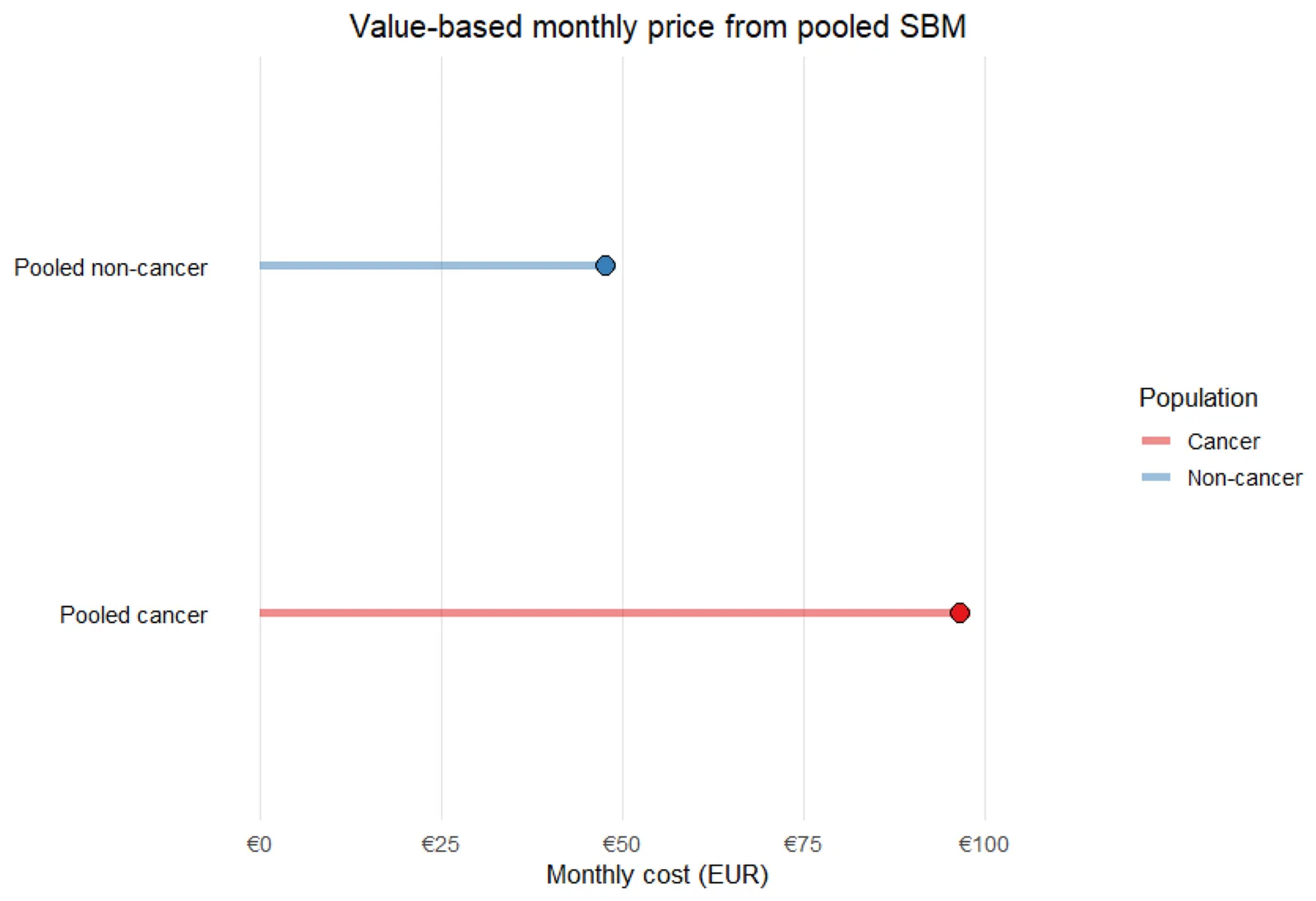
Value-based monthly cost estimates for PAMORAs derived from the pooled analysis of spontaneous bowel movement (SBM) outcomes, separately for non-cancer and cancer populations. Utilities were estimated through the two-step mapping (SBM → PAC-QOL → EQ-5D) and monetized at a willingness-to-pay threshold of €30,000 per QALY. Results are expressed per 28–30 days of therapy. Blue indicates the non-cancer population, and red the cancer population.

**Table 1:** Characteristics of included randomised controlled trials.

Study/Year	PAMORA Drug & Dose	Population Setting	Sample Size (Intervention/Control)	Age (Years) */Median (IQR)	Sex (% Female) *	Baseline Opioid Exposure (MEDD mg/Day) *	Follow-Up Duration
Hale, 2017 (COMPOSE-1) [28]	Naldemedine 0.2 mg	Non-Cancer	273/272	53.0 (47.0–60.0)/53.0 (46.0–60.5)	59.0%/62.0%	108.1/128.4	12 weeks
Hale, 2017 (COMPOSE-2) [28]	Naldemedine 0.2 mg	Non-Cancer	276/274	54.0 (47.5–61.0)/54.0 (47.0–60.0)	60.0%/61.0%	106.9/113.2	12 weeks
Webster, 2018 (COMPOSE-3) [27]	Naldemedine 0.2 mg	Non-Cancer	621/619	53.4 ± 11.7/52.7 ± 10.6	61.7%/64.9%	123.0 ± 146.1/121.2 ± 163.4	52 weeks
Katakami, 2018 (COMPOSE-4) [33]	Naldemedine 0.2 mg	Cancer	97/96	63.8 ± 9.4/64.6 ± 11.8	39.2%/37.5%	57.3 ± 46.4/69.5 ± 99.5	2 weeks
Katakami, 2017 [32]	Naldemedine 0.2 mg	Cancer	58/56	63.4 ± 10.4/64.2 ± 9.6	41.4%/39.3%	82.3 ± 87.2/85.5 ± 98.5	2 weeks
Webster, 2017 [31]	Naldemedine 0.2 mg	Non-Cancer	59/61	50.7 ± 11.4/53.1 ± 10.9	64.4%/73.8%	124.3 ± 158.6/146.5 ± 212.5	12 weeks
Hamano, 2024 [17]	Naldemedine 0.2 mg	Cancer	48/47	67.8 (33–84)/ 66.4 (32–85)	51%/68.0%	Oxycodone 85.7%/88.0%*	2 weeks
Chey, 2014 (KODIAC-04) [18]	Naloxegol 25 mg	Non-Cancer	212/211	52.2 ± 10.3/52.9 ± 10.0	55.1%/65.4%	143.2 ± 150.1/135.6 ± 145.8	12 weeks
Chey, 2014 (KODIAC-05) [18]	Naloxegol 25 mg	Non-Cancer	226/231	51.9 ± 12.1/52.3 ± 11.6	63.4%/62.5%	136.4 ± 134.3/119.9 ± 103.8	12 weeks
Paulson, 2005 [23]	Alvimopan 0.5 mg	Non-Cancer	56/54	51.5 ± 1.5/48 ± 1.2	55.3%/64.8%	111.2 (10–1500)/85 (10–597)	12 weeks
Irving, 2011 [25]	Alvimopan 0.5 mg (QD/BID)	Non-Cancer	160/164	52.1 ± 11.6/52.3 ± 11.5	64.5%/62.8%	Nr	12 weeks
Michna, 2011 [26]	Methylnaltrexone (QD/QOD)	Non-Cancer	150/162	48.3 (23–78)/49.7 (25–83)	59.7%/61.1%	157.9 (7.2–1334.3)/160.8 (13.6–1286.5)	12 weeks
Webster, 2013 [22]	Naloxegol (5/25/50 mg)	Non-Cancer	130/129	50.6 ± 11.9/48.8 ± 11.6	61.1%/63.2%	58.9%/56.8%	12 weeks
Jansen, 2011 [24]	Alvimopan 0.5 mg (QD/BID)	Non-Cancer	172/172	51.5 ± 11.2/52.1 ± 11.3	60.1%/69.2%	Nr	12 weeks
Singla, 2012 [29]	Bevenopam (0.1/0.25 mg BID)	Non-Cancer	45/43	50	nr	248–273	4 weeks
Vickery, 2011 [20]	Axelopran (0.25–10 mg QD)	Non-Cancer	70/54	Nr	nr	Nr	12 weeks
Portenoy, 2008 [34]	Methylnaltrexone (1–20 mg)	Cancer	23/10	61.1 ± 19.0	55.0%	180.0 (range 9–1207)	Nr
Bull, 2015 [35]	Methylnaltrexone 8/12 mg QOD	Cancer	116/114	65.3 ± 12.9/65.7 ± 13.0	48.3%/49.1%	180.0 (4.5–4427.0)/160.8 (9.0–7228.6)	2 weeks
Ross, 2012 [21]	Axelopran (0.25–10 mg QD)	Non-Cancer	217/55	51.8 ± 10.4/50.9 ± 10.3	64.9%/66.2%	155.0 (27.0–2289.3)/141.1 (30.0–1280.0)	12 weeks

* exact continuous baseline MEDD was not reported; 85.7% (intervention) and 88.0% (control) of patients were receiving oxycodone at enrollment.

**Table 2:** Conversion table PAC-QOL ↔ EQ-5D.

Study	Population	Instrument	Mean Δ (vs. Placebo)
Hamano, 2024 [17] (Naldemedine)	Cancer	PAC-QOL: −0.49 EQ-5D-5L: +0.04	≈0.5 PAC-QOL point ≈ 0.04 EQ-5D → 1 PAC-QOL point ≈ 0.08 EQ-5D
KODIAC-04 (Naloxegol) [18]	Non-cancer	PAC-QOL: −0.28 EQ-5D-3L: +0.03	≈0.3 PAC-QOL point ≈ 0.03 EQ-5D → 1 PAC-QOL point ≈ 0.10 EQ-5D
KODIAC-05 (Naloxegol) [18]	Non-cancer	PAC-QOL: −0.18 EQ-5D-3L: +0.02	≈0.2 PAC-QOL point ≈ 0.02 EQ-5D → 1 PAC-QOL point ≈ 0.10 EQ-5D

## Data Availability

The original contributions presented in this study are included in the article/Supplementary Materials. Further inquiries can be directed to the corresponding author.

## Supplementary Materials

The following supporting information can be downloaded at: https://www.mdpi.com/article/10.3390/jpm16100494/s1.
