# Supplementary material for: PAMORAs in Cancer and Non-Cancer Opioid-Induced Constipation: A Systematic Review, Meta-Analysis, and Personalized Value-Based Evaluation Integrating Clinical and Patient-Reported Outcomes
Source: J Pers Med. 2026 Sep 24;16(10):494. doi: 10.3390/jpm16100494 (PMC13705715; doi:10.3390/jpm16100494)
Supplement: Supplementary file 1 [file jpm-16-00494-s001.zip › jpm-4459238-supplementary.pdf]

Supplementary Table S1. Full reproducible search strategy across databases.

Database	Search Strategy
**PubMed / MEDLINE**	(("Peripherally Acting Mu-Opioid Receptor Antagonists"[Mesh] OR "PAMORA" OR "naldemedine" OR "methylnaltrexone" OR "alvimopan" OR "naloxegol" OR "bevenopran" OR "axelopran") AND ("Opioid-Induced Constipation"[Mesh] OR "OIC") AND ("Randomized Controlled Trial"[ptyp] OR "randomized" OR "placebo"))
**Embase**	('peripherally acting mu opioid receptor antagonist'/exp OR 'PAMORA':ti,ab OR 'naldemedine':ti,ab OR 'methylnaltrexone':ti,ab OR 'alvimopan':ti,ab OR 'naloxegol':ti,ab OR 'bevenopran':ti,ab OR 'axelopran':ti,ab) AND ('opioid induced constipation'/exp OR 'OIC':ti,ab) AND ('randomized controlled trial'/exp OR 'placebo'/exp)
**Cochrane (CENTRAL)**	("PAMORA" OR "naldemedine" OR "methylnaltrexone" OR "alvimopan" OR "naloxegol" OR "bevenopran" OR "axelopran") AND ("opioid-induced constipation" OR "OIC") in Title Abstract Keyword

Table S2. Assessment of Risk of Bias for Included Randomized Controlled Trials

Study	Drug	Population	Random Sequence Generation	Allocation Concealment	Blinding (Participants/Personnel)	Blinding (Outcome Assessment)	Incomplete Outcome Data	Selective Outcome Reporting	Other Bias	Overall Risk
**Compose 1**	Naldemedine	Non-cancer	Low	Low	Low	Low	Low	Low	High	Low
**Compose 2**	Naldemedine	Non-cancer	Low	Low	Low	Low	Low	Low	High	Low
**Compose 3**	Naldemedine	Non-cancer	Low	Low	Low	Low	Low	Low	High	Low
**Compose 4**	Naldemedine	Cancer	Low	Low	Low	Low	Low	Low	High	Low
**Katakami 2017**	Naldemedine	Cancer	Low	Low	Low	Low	Low	Low	Unclear	Low
**Webster (2017)**	Naldemedine	Non-cancer	Low	Low	Low	Low	Low	Low	Unclear	Low
**Hamano**	Naldemedine	Cancer	Low	Low	Low	Low	Low	Low	Low	Low
**Bull**	Methylnaltrexone	Cancer	Low	Unclear	Low	Low	Low	Low	High	Low
**Michna**	Methylnaltrexone	Non-cancer	Low	Low	Low	Low	Low	Low	High	Low
**Portenoy**	Methylnaltrexone	Cancer	Low	Unclear	Low	Low	Unclear	Low	Unclear	Unclear
**Irving**	Alvimopan	Non-cancer	Low	Low	Low	Low	Low	Low	High	Low
**Jansen**	Alvimopan	Non-cancer	Low	Low	Low	Low	Low	Low	High	Low

Study	Drug	Population	Random Sequence Generation	Allocation Concealment	Blinding (Participants/Personnel)	Blinding (Outcome Assessment)	Incomplete Outcome Data	Selective Outcome Reporting	Other Bias	Overall Risk
**Paulson**	Alvimopan	Non-cancer	Low	Low	Low	Low	Low	Low	Unclear	Low
**Webster (2013)**	Naloxegol	Non-cancer	Low	Low	Low	Low	Unclear	Low	High	Low
**KODIAC-04**	Naloxegol	Non-cancer	Low	Low	Low	Low	Low	Low	Low	Low
**KODIAC-05**	Naloxegol	Non-cancer	Low	Low	Low	Low	Low	Low	Low	Low
**Singla**	Bevenopran	Non-cancer	Low	Unclear	Low	Low	Unclear	Low	Low	Low
**Vickery**	Axelopran	Non-cancer	Low	Unclear	Low	Low	Low	Low	Unclear	Unclear
**Ross**	Axelopran	Non-cancer	Low	Unclear	Low	Low	Low	Low	Unclear	Unclear

Figure S1.

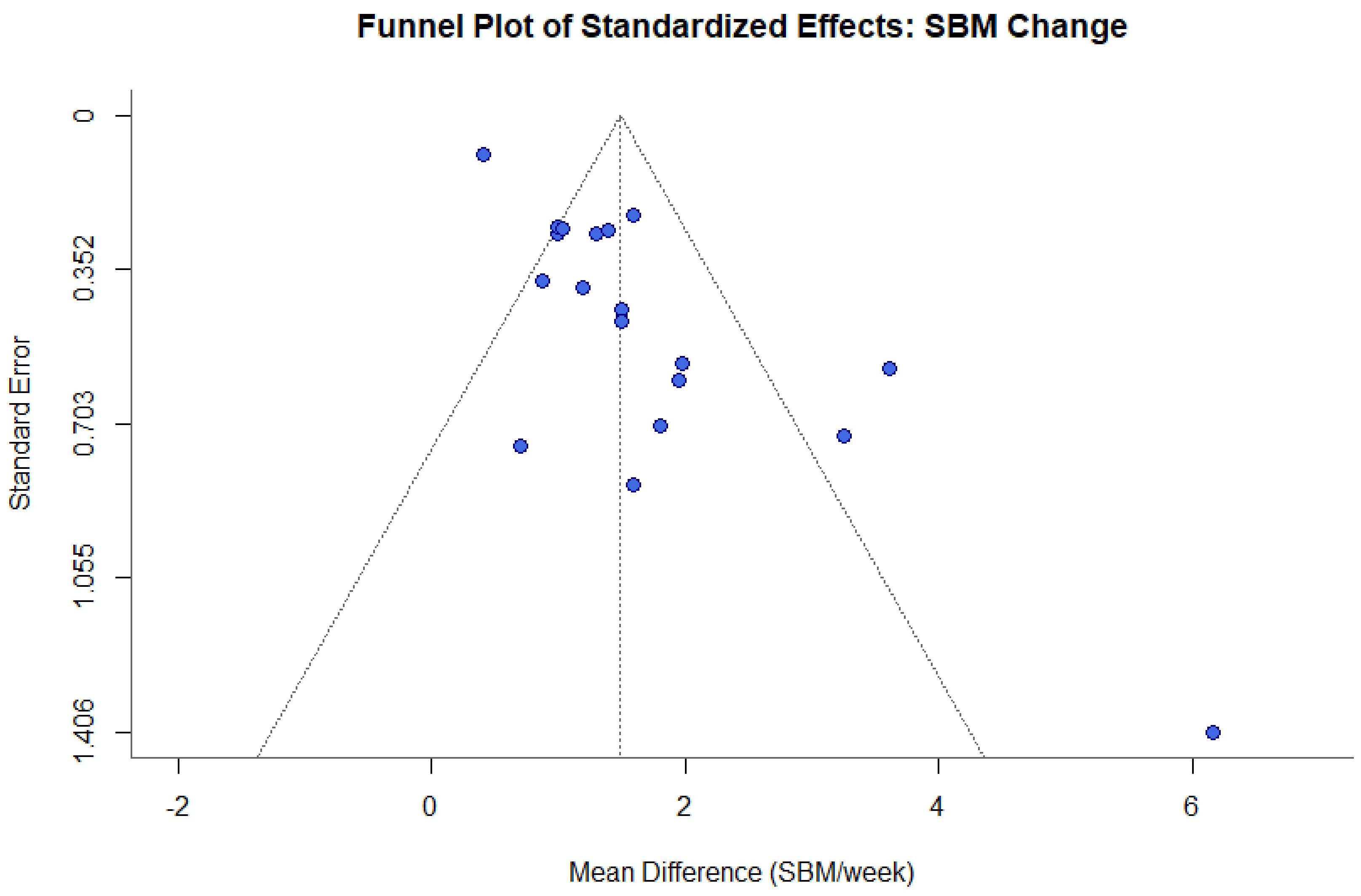

Funnel Plot of Standardized Effects: SBM Change
Standard Error
0
0.352
0.703
1.055
1.406
-2
0
2
4
6
Mean Difference (SBM/week)

Figure S2.

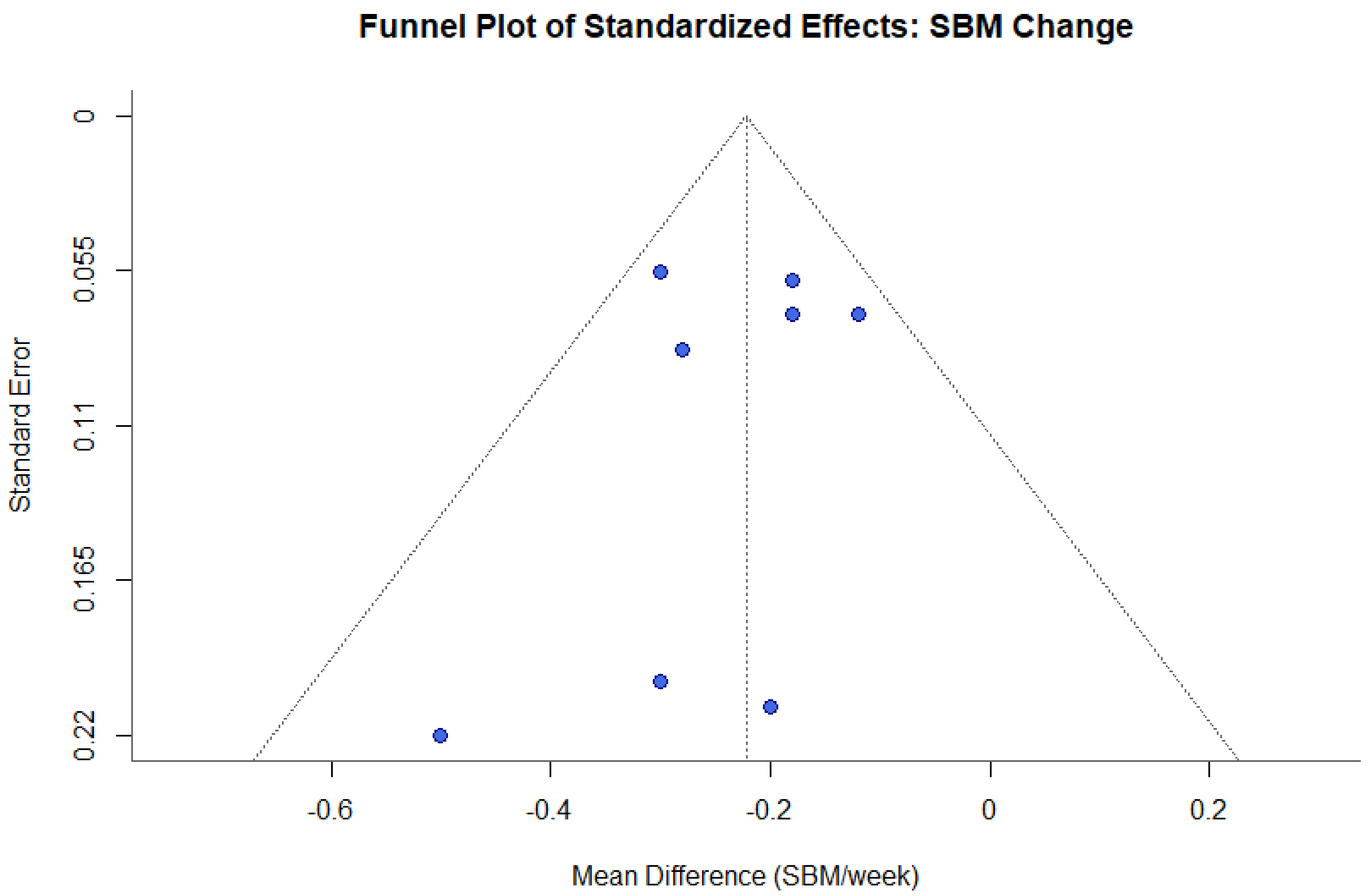

Funnel Plot of Standardized Effects: SBM Change
Standard Error
0
0.055
0.11
0.165
0.22
-0.6
-0.4
-0.2
0
0.2
Mean Difference (SBM/week)
